# A new radiological classification of the mental foramen in the edentulous mandible: clinical and surgical implications

**DOI:** 10.1007/s10006-026-01623-8

**Published:** 2026-08-27

**Authors:** Bruno Scognamiglio, Alfonso Acerra, Mario Caggiano, Isabella De Rubertis, Francesco Giordano

**Affiliations:** 1 Department of Medicine, Surgery and Dentistry, University of Salerno, Via Salvador Allende, 43, Baronissi, Salerno 84081 Italy; 2 Department of Medical Biotechnologies, Unit of Periodontology, University of Siena, Siena, Italy

**Keywords:** Mental foramen, Mental nerve emergence, Edentulous mandible, CBCT scans, Oral surgery, Implant dentistry, Flap design

## Abstract

**Background:**

The purpose of this cross-sectional study was to classify different patterns of mental nerve emergence (MNE) in edentulous mandible using cone beam computed tomography (CBCT) scans.

**Methods:**

Seventy-five consecutive patients, each contributing with one hemimandible and candidates for implant surgery, were recruited at Dental Unit of University Hospital of Salerno. CBCT scans were analyzed by two calibrated examiners to assess the distance between the bone crest and the mental foramen (BC–MF), mandibular bone resorption according to the Cawood–Howell (C-H) classification and the pattern of MNE. Inter-rater agreement was evaluated using weighted Cohen’s kappa. Descriptive statistics were calculated and non-parametric tests were used to compare BC–MF distances among C-H categories and MNE classes, as well as to assess associations between categorical variables.

**Results:**

Significant differences were observed among MNE-Classes with significantly higher BC–MN distance in Class I compared with Classes II and III, and in Class II compared with Class III (*p* < 0.05). Moreover, a statistically significant association between the MNE class and the C-H classification (*p* < 0.001) was highlighted.

**Conclusions:**

Soft tissue management in the edentulous mandible requires particular attention to reduce the risk of MN injury, especially in MNE Class II and III. A statistically significant association was observed between MNE patterns and C-H classes, with higher emergence classes associated with a reduced BC-MF distance. The proposed radiological classification may support preoperative surgical planning by facilitating the identification of different patterns of MNE.

## Introduction

The mental nerve (MN) is the larger terminal branche of the inferior alveolar nerve [1]. It exits through the mental foramen (MF) of the lower jaw and it is distributed to the skin of the chin and lower lip [1]. The mental foramen is the end of the mandibular canal and it is usually one for each emi-arch, but it can be also multiple and in some cases anatomical variations with four foramina at each side have been described [2]. The location of the foramen has a huge variability depending in a lot of different factors such as the age of the patient or the presence or the absence of the teeth [2].

Tooth loss in fact leads to remodeling of both hard and soft tissues, resulting in the changes of the residual bone volume [3, 4]. Most of this resorption, which varies from subject to subject, occurs in the first three-six months after tooth loss [5, 6]. Specifically, the resorption pattern has a first phase of vertical volumetric reduction and a second horizontal phase [7, 8]. In 1988 Cawood and Howell described a predictable anatomical pattern of resorption of the edentulous jaw [9]. As highlighted by Cawood and Howell, in the IV, V and VI mandible (anterior and posterior) classes the distance between the superior border of the mandible and mental foramina is progressively reduced, until the point in which it is equal to zero (the mental foramen is located on the most cranial bone position) [9].

Yosue and Brooks studied the difference of the mental foramen detection on panoramic and periapical radiographs and concluded that the mental foramen appears more consistently on panoramic than on periapical radiographs and presumed the radiographic landmark called “mental foramen” on panoramic or periapical radiographs is not the true mental foramen, but rather a portion of the mental canal after it leaves the mandibular canal [10]. For this type of evaluation CT scans are better than traditional radiographs. To date CBCT is the most precise and reliable method to assess the position of the mental foramen in the everyday clinical practice [11]. A review by Pelè et al. conducted through evaluation on CBCT scans concluded that the mental foramen was located mostly between the two premolars (between 50.4% and 61.95%) or apically to the second premolar (from 50.3% to 57.9%) [12]. In addition Artas and Yalcin evaluated also the reliability of an ultrasonographic evaluation of the mental foramen and of the blood flow of the mental artery and concluded that the CBCT is the gold standard and the US is not as effective as CBCT [13].

The relevance of pre-operative investigation of the position of the mental foramen, therefore of its management arises from the chance of having nerve injury during oral surgery procedures such as implant insertion [14]. Pre-operative planning of implant surgery in the premolar region must in fact take into account the particular anatomy of the mental nerve, which, emerging from the foramen makes an anterior loop [15]. However, the intra-operative management of the loop’s position is not the only aspect of concern regarding the mental nerve. Comparable attention must be directed toward the mental nerve during the soft tissue management, especially in the incision phase of the surgery. The aim of this study is to identify different patterns of emergency of the mental nerve in the edentulous mandible and to propose a radiological classification based on CBCT evaluation.

## Materials and methods

### Study design

The present cross-sectional study was written based on the Strengthening the Reporting of Observational Studies in Epidemiology (STROBE) guidelines [16]. The study was conducted in accordance with Declaration of Helsinki. All enrolled patients provided written informed consents with permission to use their data for scientific purpose.

### Setting and participants

For the purpose of this cross-sectional study, patients were recruited at Dental Unit of the San Giovanni di Dio and Ruggi d’Aragona University Hospital of Salerno from December 2024 to June 2025. Patients had to fulfill the following inclusion criteria: (a) patients aged 18 or older; (b) at least one edentulous hemi-mandible; (c) candidates for implant surgery; (d) patients with ASA 1 or 2. Exclusion criteria were as follows: (a) poor image quality; (b) presence of teeth in the hemi-mandible; c) patients with the following medical conditions: irradiated patients in the head/neck district in the previous 6 months, immunodeficient or immunosuppressed patients, treated or under treatment with intravenous amino-biphosphonates, active infection or severe inflammation in the area intended for implant placement d) pregnant or breast feeding patients. Eligible patients were screened based on clinical and CBCT examination.

### CBCT variables

Two calibrated operators (A.A. and B.S.) performed the CBCT measurements. Calibration was carried out using five repeated measurements on five CBCT examinations not included in the study sample (weighted Cohens kappa 0.95). After the initial screening phase, sagittal sections of the acquired CBCTs were evaluated. Specifically, the distance between the mental foramen (MF) and the bone crest (BC), the severity of basal mandibular bone resorption and the pattern of mental nerve emergence (MNE) were assessed.

#### BC-MF distance

The slice used for the study measurements was the one where the maximum diameter mesio-distally of the mental foramen was appreciated. Therefore, the distance between the bone crest and the mental foramen (the most cranial point) (BC- MF distance) was measured.

#### Basal mandibular bone resorption classification

The condition of physiological remodeling of bone tissue after tooth loss has been described by Cawood and Howell according to a predictable model [9]. This classification describes five classes of atrophy for the upper jaw and six classes for the lower jaw. Specifically:

- Class I: teeth are in place;
- Class II: immediate post-extraction alveolar ridge;
- Class III: late post-extraction alveolar ridge with re-ossification of the post-extraction socket and rounded alveolar process but adequate in height and thickness;
- Class IV: ridge with adequate height but insufficient thickness, defined as “knife-edge”;
- Class V: flat ridge, inadequate in both height and thickness;
- Class VI: (only for the lower jaw) depressed ridge, with atrophy of the basal bone.

#### MNE pattern

**Fig. 1:**
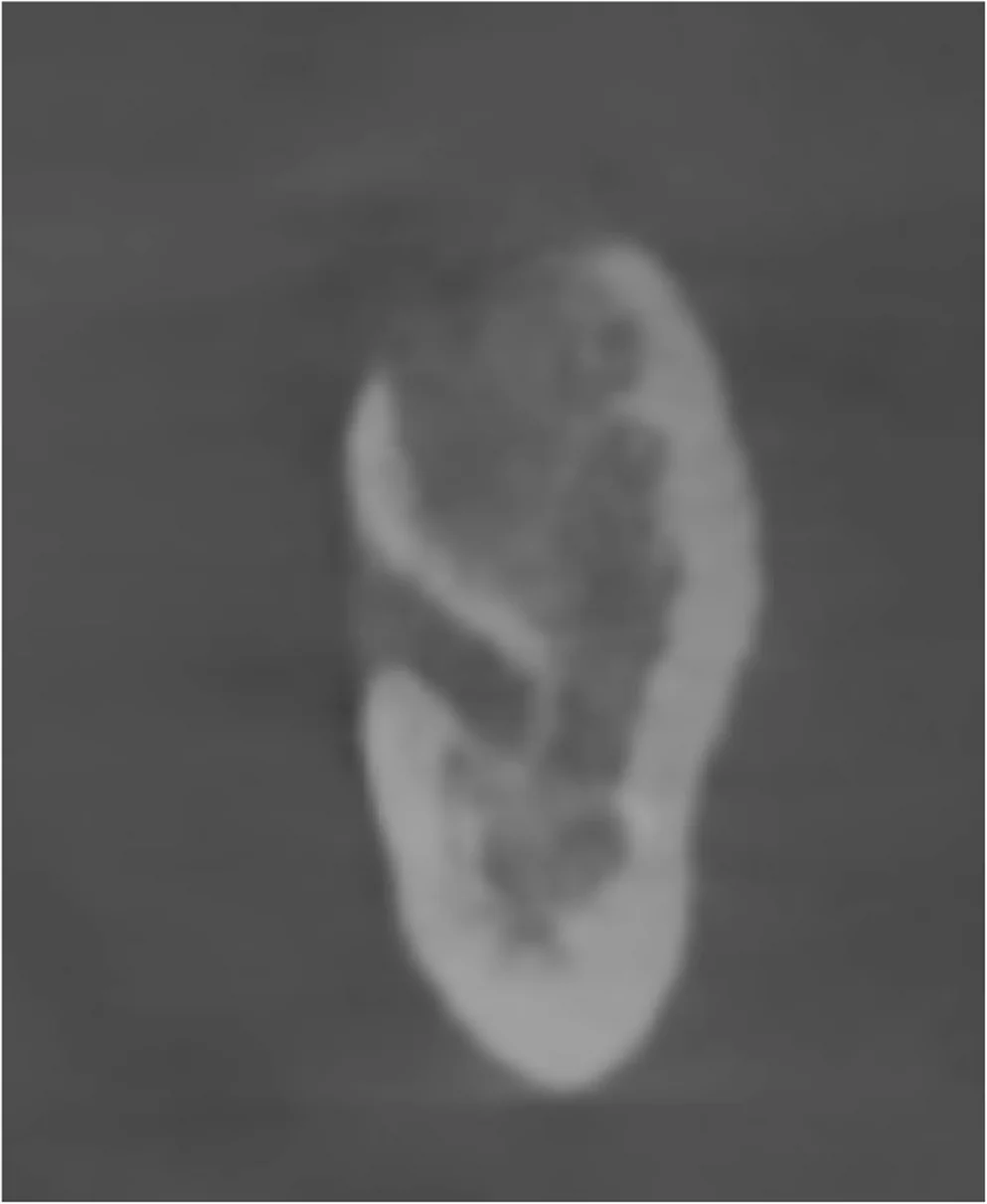
MNE - class 1. MNE = mental nerve emergence

**Fig. 2:**
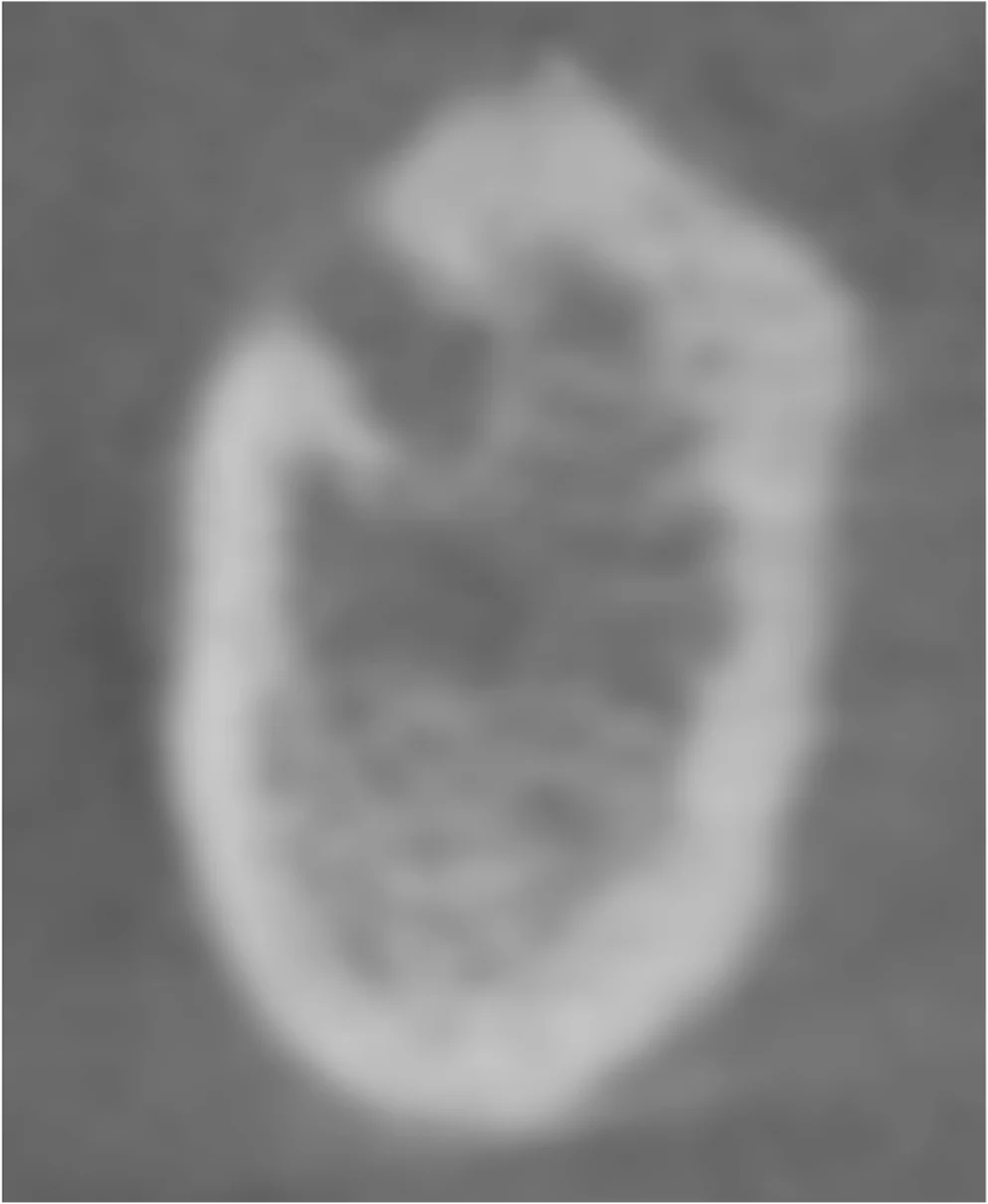
MNE - class 2. MNE = mental nerve emergence

**Fig. 3:**
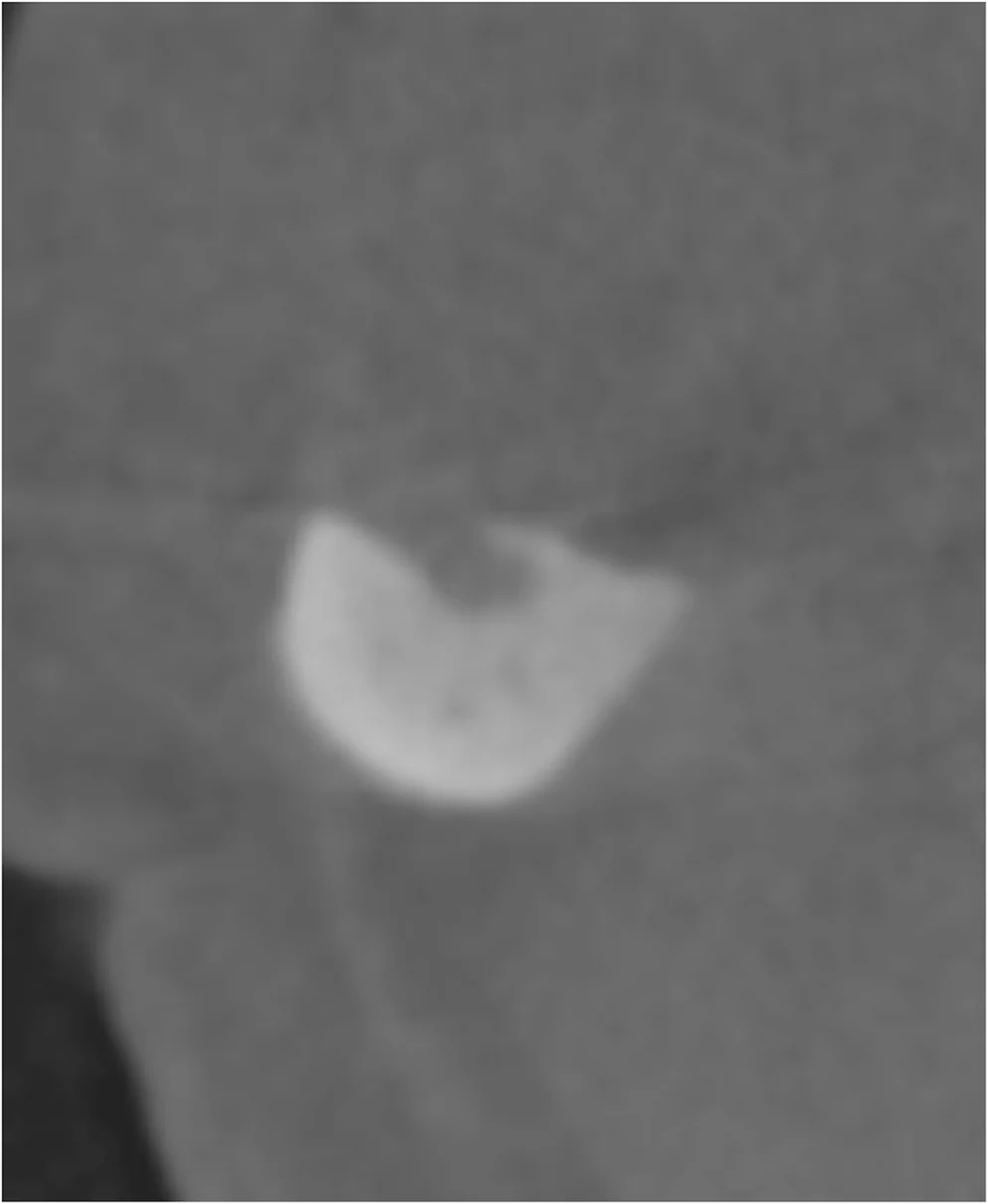
MNE - class 3. MNE = mental nerve emergence

Three different patterns of MNE were detected:

- **MNE-Class 1**: The mental foramen is located on the vestibular side of the jaw and emerges from a vertical buccal wall; bone resorption does not involve the mental foramen roof. [Fig. 1]

- **MNE-Class 2**: The resorption of the mental foramen roof is partial, leaving a residual bone ridge on the lingual side, MN emerges with an horizontal trajectory. [Fig. 2]

- **MNE-Class 3**: The resorption of the mental foramen roof is complete, the mental foramen is located on the most cranial part of the mandible, emerging horizontally. [Fig. 3]

### Data sources

CBCT scans with standardized settings were acquired using Carestream Dental LLC, Atlanta, USA, with the following acquisition parameters (120 kV, 6.3 mA, 20 s, 150 μm isotropic voxel size, slice interval distance 150 μm, FOV 10 × 10) with the teeth separated with a cotton roll. The scans were evaluated using Carestream CS Imaging 8.0.27, Atlanta, USA, in both sagittal view and 3D reconstruction.

### Sample size

Given the exploratory nature of the study, the sample size was pragmatically determined and consisted of 75 consecutive patients referred during the recruitment period. The present study should therefore be considered a pilot study.

### Statistical methods

Statistical analyses were performed using dedicated software (STATA IC, version 18, StataCorp LP, TX, USA). Continuous data were presented as mean and standard deviation, while categorical variables were reported as counts and proportions. The normality of data distribution was assessed using the Shapiro–Wilk test. Inter-rater agreement in the assignment of mental nerve (MN) classification was evaluated with Weighted Cohen’s Kappa with linear weights (“kappaetc” command), to account for the ordinal nature of the classification. Differences in the BC-MF distance among Cawood-Howell (C-H) categories and among MNE classes were analyzed using the Kruskal-Wallis test, followed by Dunn’s post-hoc correction.

Associations between categorical variables (MNE class vs. C-H category) were examined using the Chi-square test. A p-value < 0.05 was considered statistically significant.

## Results

A total of 77 patients, each contributing with one hemimandible, were screened for inclusion. Two experimental sites were excluded due to poor image quality. Consequently, 75 edentulous hemimandibles were considered suitable for analysis (27 males, 48 females). At the time of the radiographic examination, the mean patient age was 67.23 ± 8.41 years.

Table 1 presents the frequency distribution of the assignment of each experimental site according to the Cawood-Howell classification and the MNE class, as well as the overall mean BC–MF distance.

**Table 1:** Frequency distribution of Cawood-Howell classification, MNE class, and overall BC–MF distance

Cawood-Howell classification [*N*, (%)]	
Class III	9 (12)
Class IV	8 (10.66)
Class V	26 (34.67)
Class VI	32 (42.67)
MNE-Class [N, (%)]	
Class I	31 (41.33)
Class II	27 (36)
Class III	17 (22.67)
BC-MF distance (mm) (Mean ± SD)	
	3.49 ± 2.79

Abbreviations: *MNE* Mental Nerve Emergence, *BC-MF* bone crest-mental foramen

**Fig. 4:**
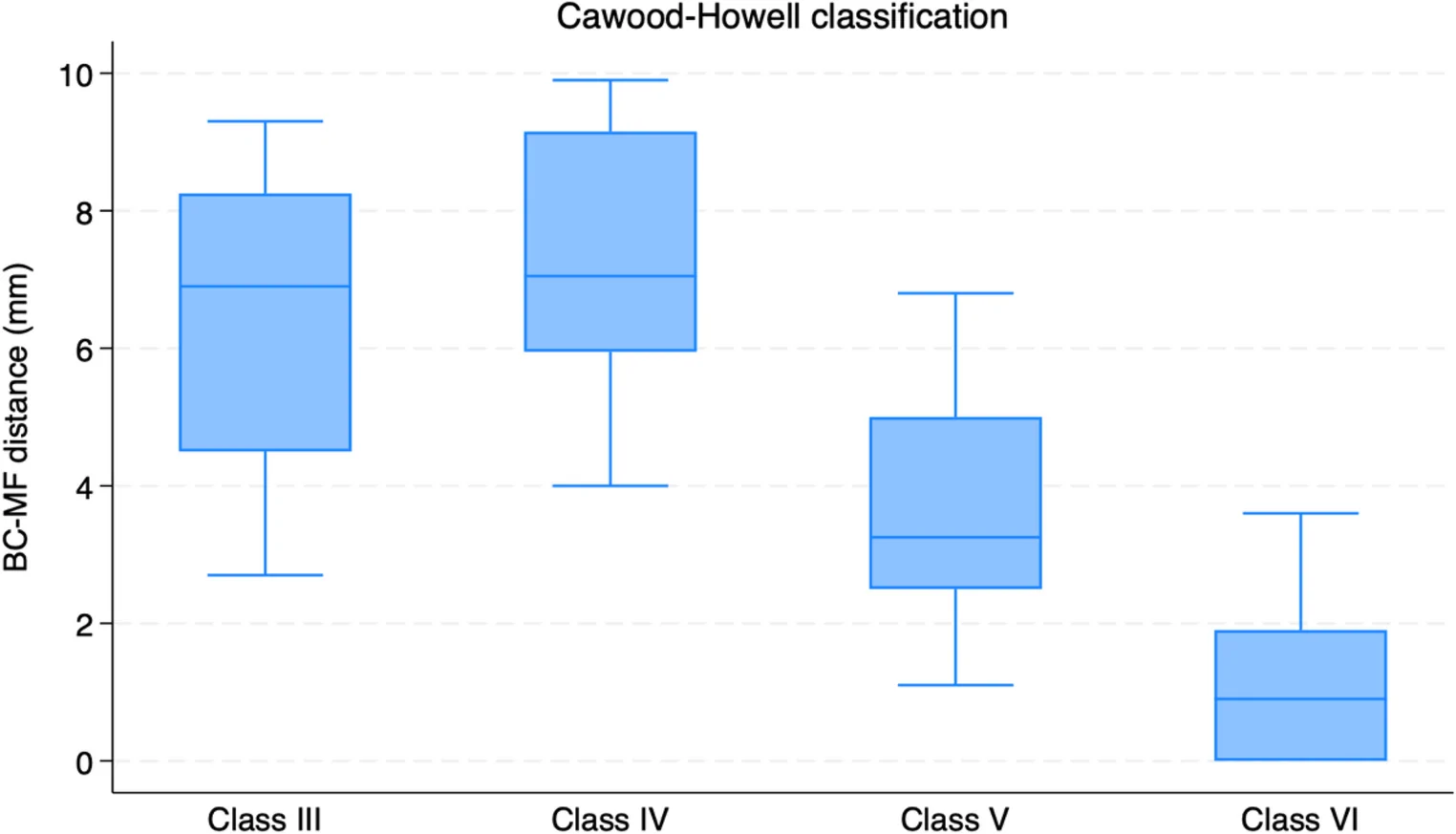
Distribution of BC–MN distance across C-H classes. BC-MF = bone crest-mental foramen; C-H = Cawood-Howell

**Fig. 5:**
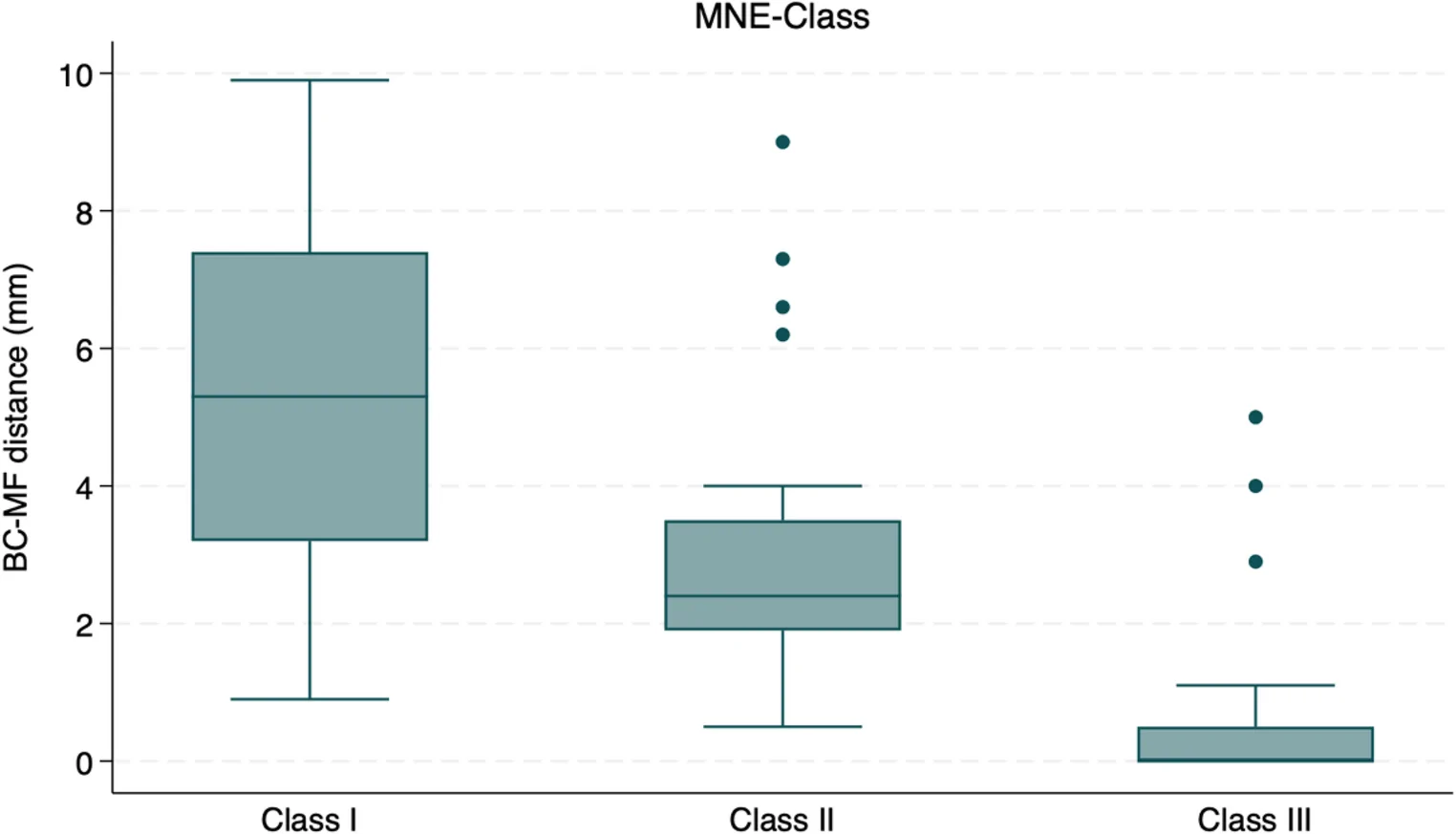
Distribution of BC–MN distance across MNE classes. BC-MF = bone crest-mental foramen; MNE = Mental Nerve Emergence

Almost perfect inter-rater agreement was observed between the two independent examiners (B.S. and A.A.), with a weighted Cohen’s kappa of 0.9502 and an overall percentage agreement of 98.03%. The box plots in Figs. 4 and 5 display the distribution of BC–MN distance across Cawood and Howell classes and MNE classes, respectively, showing a progressive reduction in BC-MN distance with increasing classes.

Post-hoc Dunn’s test showed significant differences in BC-MN distance between Cawood–Howell Class IV and V (*p* = 0.007), and between Class VI and all other classes (*p* < 0.001). Significant differences were observed among MNE-Class with significantly higher BC–MN distance in Class I compared with Classes II and III, and in Class II compared with Class III (all *p* < 0.001) (Table 2).

**Table 2:** Mean BC-MF distance across Cawood–Howell and MNE classification classes

	MNE Class I	MNE Class II	MNE Class III	
	5.35 ± 2.53*	3.02 ± 2.02*	0.81 ± 1.58*	
BC-MF distance (mm) (Mean ± SD)	C-H Class III	C-H Class IV	C-H Class V	C-H Class VI
	6.42 ± 2.29*	7.26 ± 2.05*	3.7 ± 1.58*	1.03 ± 1.07*

Abbreviations: *MNE* Mental Nerve Emergence, *C-H* Cawood-Howell, *BC-MF* bone crest-mental foramen

*p-Value < 0.05 for inter-class comparisons

A cross-tabulation was performed to examine the relationship between the two classifications of the MN. Categories distribution is shown in Table 3. The Pearson chi-square test indicated a statistically significant association between the two classifications (χ² = 35.53, *p* < 0.001).

**Table 3:** Relationship between Emi-mand and Cawood-Howell classifications of the MN

	Cawood-Howell III	Cawood-Howell IV	Cawood-Howell V	Cawood-Howell VI	Total
MNE-Class I	9 (100%)	5 (62.50%)	15 (57.59%)	2 (6.25%)	31 (41.33%)
MNE-Class II	-	3 (37.50%)	11 (42.31%)	13 (40.62%)	27 (36%)
MNE-Class III		-	-	17 (53.13%)	17 (22.67%)
Total	9 (100%)	8 (100%)	26 (100%)	32 (100%)	75 (100%)

## Discussion

The present cross-sectional CBCT study provides a novel radiological classification of mental nerve emergence in edentulous mandibles. Nearly half of the analyzed hemimandibles exhibited anatomies potentially associated with an increased surgical risk during flap incision. A progressive reduction in BC-MF distance was observed with increasing Cawood-Howell classes and across MNE classes. Furthermore, a strong statistical association between the proposed MNE classification and the Cawood-Howell classification was demonstrated.

From the analysis of these data, it can be observed that in 22.67% of edentulous hemimandibles, the mental nerve emerges directly at the bone crest, while in 36% it emerges, following an horizontal trajectory, at the vestibular side of the mandible, where the mental foramen roof is partially resorbed but with a residual amount of alveolar bone still present on the lingual side. In these cases, particularly in Class 3, the surgical risk of nerve injury during flap incision and elevation could be increased.

Analysis of the different patterns of MNE showed that Class 1 represents a low-risk condition for nerve injury during flap design, as the mental nerve emerges buccally, with preserved anatomical separation from the bone crest. In this anatomical configuration, it is advisable to place the incision in the keratinized tissue whenever possible.

Class 2, reflects an intermediate condition, characterized by partial resorption of foramen bone roof and reduced bony protection of the mental nerve. In this scenario, residual alveolar bone may still serve as a partial anatomical landmark for determining the incision site, although its reliability decreases with increasing ridge resorption. This condition requires increased caution, due to the closer relationship between mental nerve and bone crest.

Class 3 represents the most advanced condition, characterized by complete resorption of mental foramen roof. In this scenario anatomical landmarks are markedly reduced, making pre-operative radiographic evaluation essential for surgical planning. This pattern is associated with a higher potential risk of mental nerve injury due to the absence of protective bony structures.

This assessment is pivotal for appropriate and accurate soft tissue management in the surgery of edentulous mandible. The proposed approaches are specifically designed to reduce the risk of nerve injury during the initial phase of flap preparation.

Nerve injuries can be painful and significantly impact the patient’s quality of life [17]. The severity of nerve damage determines the associated complications, which can range from transient hypoesthesia to neuropathic pain or even trigeminal neuralgia [18]. A damage of the mental nerve during flap incision may result in sensory disturbances, numbness, tingling, or pain in the lower lip, chin, or gingiva [19]. In some cases, nerve damage may lead to long-term or permanent sensory deficits, potentially becoming the subject of medico-legal disputes [20]. When surgical procedures are performed in areas close to critical anatomical structures, failure to meticulously assess the radiographic examination and make appropriate clinical decisions could not only expose the clinician to legal liability, but could also be considered a breach of professional duty. In such cases, nerve damage is more easily classified as preventable rather than as an intrinsic surgical complication. The choice of flap design and the extent of soft tissue elevation are clinical decisions that must be based on accurate anatomical assessment. Therefore, inadequate planning or inappropriate choice of incision may constitute negligent or imprudent conduct. Furthermore, inadequate preoperative informed consent regarding risks may result in liability regardless of technical performance, particularly when sensory disturbances persist or become permanent. Consequently, careful radiological evaluation and tailored surgical planning are not only clinical requirements but also essential safeguards for reducing potential medico-legal consequences.

### Limitations of the study

The study design and the inclusion of patients from a single center may limit the generalizability of the findings. Although CBCT-based measurements demonstrated excellent inter-rater agreement, the proposed classification was developed based on radiological parameters and has not yet been prospectively validated as a clinical decision-making tool in surgical planning.

## Conclusion

This study highlights the importance of careful pre-operative assessment of MNE and soft tissue management in the edentulous mandible to minimize the risk of mental nerve injury during surgical procedures. Higher MNE classes were associated with a progressively reduced BC-MF mean distance and showed a statistically significant correlation with C-H classes, supporting the relationship between increasing mandibular resorption and greater surgical complexity. The proposed radiological classification may represent a useful adjunct for risk stratification and flap design, facilitating tailored surgical planning for edentulous mandible. Nevertheless, further prospective clinical studies are warranted to validate its reproducibility and clinical applicability.

## Data Availability

The data supporting the findings of this study are not publicly available due to ethical restrictions but are available from the corresponding author on reasonable request.
